# New Views of Chronic Bright's Disease

**Published:** 1913-06-28

**Authors:** 


### NEW VIEWS OF CHRONIC BRIGHT'S DISEASE.

### Functional Tests as a Basis of Classification.

For many years cases of chronic Bright's disease were correlated in the text-books with the different varieties of kidney disease found at *post-mortem* examinations. In other words, morbid anatomy was the basis of classification, and physicians spent much time and trouble in making up their minds whether their patients were suffering from large white or small red kidney, granular or arteriosclerotic kidney, and so on. As a matter of fact it not at all rarely happened that the signs and symptoms relied on to decide this pathological diagnosis proved to be quite fallacious when a case was traced as far as the *post-mortem* room; and even the most experienced found themselves frequently wrong in their prognostications about the exact size and colour of the kidneys removed from fatal cases of Bright's disease.

Eventually it began to dawn on men of original views that the old classification was unsatisfactory, and of late years there has been a tendency to break away from morbid anatomy and to rely much more on clinical classification as a basis of diagnosis, prognosis, and treatment. In a paper read before the Clinical Society of the New York Post-Graduate Medical School Dr. H. E. Baright * propounds a scheme based upon experimental pathology.

### Vivisectional Foundations.

To start with, two quite separate clinical and pathological types of nephritis can be produced in animals by poisoning them with cantharides and corrosive sublimate respectively. These types are constant; that is, the same drug always produces its own type of nephritis and not the other type. The correlation of these experimentally produced kinds of nephritis with known clinical types in man is not as yet complete, but it is being accomplished by what is known as the method of functional testing.

This method is, briefly, as follows: Certain comparatively harmless substances are administered to healthy animals, and the time required for their complete elimination in the urine is noted. The same substances are then administered to animals suffering from experimental nephritis of different types, and the time of elimination again ascertained. The excretion time of these substances is then tested on human beings, both in normal health and when suffering from clinical nephritis.

Thus it has been found that the excretion of lactose (milk-sugar) is delayed in human beings in conditions which correspond with experimental nephritis of the cantharides type, but not in other kinds. Similarly, potassium iodide and sodium chloride are delayed in clinical conditions which correspond with experimental nephritis of the sublimate type, but not in other kinds. Urea, again, is delayed in elimination only when there is a tendency to uræmia; this tendency may co-exist with the other types of nephritis, but apparently also it may occur as a pure type, the experimental analogue and histopathology of which are as yet unknown.

* *Medical Record*, April 19, 1913, and *The Post-Graduate*, April, 1913, p. 317.

### Nephritis with Delayed Lactose Excretion.

This type of nephritis occurs pure—that is, with normal iodide and normal urea excretion—in 40 to 50 per cent. of all cases of nephritis. Pathologically it is characterised by an endarteritis, not only of the glomerular vessels of the kidney, but also of the entire vascular system of the body. This is the kind of kidney disease associated *par excellence* with high blood pressure and hypertrophied left ventricle. The lactose test is capable of revealing this condition in the really incipient stage; that is, long before it has caused symptoms sufficient to lead the patient to seek advice. As Dr. Baright says, these patients have often a sense of well-being; and when they do go to a doctor it is usually for heart troubles. They are liable to hæmorrhages: in the retina, the brain, the stomach, the nose, in fact anywhere. In later stages, anginal attacks, dizziness, sleeplessness, orthopnœa, numbness of the extremities, hemianopsia, and œdema of the lungs are common symptoms. At autopsy a condition is often disclosed which has been long called the "small white" kidney; but this is not invariable, and other forms, such as the typical granular kidney, are also not unusual.

This is the form of disease characterised especially by inflammation of the epithelium of the tubules of the kidney. It is the type which results from corrosive sublimate poisoning, and occurs clinically after scarlet fever and other infectious diseases. This inflammatory process is also not infrequently super-added on to a pre-existing glomerular (vascular) nephritis. The pure type of this tubular nephritis is seen in only 5 to 7 per cent. of cases of Bright's disease. Excretion of lactose and of urea is normal; that of potassium iodide and sodium chloride delayed. Clinically œdema, albuminuria, and oliguria are prominent features; whereas increased blood pressure and cardiac hypertrophy are not marked. In the pure form uræmia seldom develops.

### Nephritis with Urea Retention.

This type is rarely met with pure—that is, unmixed with other kinds. It is a toxæmia, due presumably to the presence in the blood of non-proteid nitrogenous substances which ought to be excreted from it by the kidneys. Normally, and in the two preceding varieties of Bright's disease, not more than 50 mg. of this non-proteid nitrogen is found in 100 c.c. of blood serum. In uræmics, on the other hand, the content may rise to 100, 200, or even 300 mg. The clinical symptoms are of the familiar uræmic kind, which it is unnecessary to summarise here. This type is, as has been said, most often seen engrafted on to one of the others, and in practice such mixed cases constitute about 40 to 50 per cent. of the total.

How the Tests are Carried Out.

The functional tests upon which this new classification of Bright's disease is based require careful attention to detail if they are to be a success.

In the *lactose* test, first exclude glycosuria by the usual methods. With strict antiseptic precautions, inject intravenously 2 grams of lactose in 20 c.c. of water. This solution is previously sterilised by heating it to 80° C. on each of four successive days for an hour. Four hours after the injection the patient is to empty his bladder; the result can be discarded. Then the bladder is to be emptied every hour, or half-hour, and tested for sugar. If the test is still positive eight hours after the injection, vascular impairment is probable; if still positive after ten hours, it is quite certain.

The *iodide* test should not be employed when there is hyperthyroidism. At 5 p.m. give half a gram (seven grains) of potassium iodide by the mouth. In health this will be all got rid of in forty hours; that is by 9 a.m. of the next day but one. Delay beyond this is suspicious; beyond sixty hours, positive indication of tubular nephritis. Any of the usual tests for the presence of iodides in the urine will answer. For the *chloride* test, make the water and salt intake as nearly uniform as possible for several days, and determine the urinary output of each. Then give ten grams of sodium chloride with one litre of water, in addition to the ordinary supply. Collect the urine for several days, or as long as any excess of salt appears. Normally one litre of water and ten grams of salt should be eliminated in twenty-four hours.

For the *urea* test, determine the total nitrogen output with a uniform nitrogenous intake. Then give ten grams of urea by the mouth and collect the urine as long as any excess of urea appears; normally this quantity should be excreted in twenty-four hours.

Conclusions.

These complicated procedures are neither easy nor inexpensive; and they are much more adapted for institutional work than for private practice. This much Dr. Baright admits, but he looks forward to a simplification of the tests in the future; and he emphasises especially the merits of more exact and earlier diagnosis which these functional tests offer, and their value as guides in treatment. If experience substantiates these claims, functional testing has come to stay; and physicians will, we suppose, have to submit to seeing chronic Bright's disease become the property of a new group of specialists, just as during the last three years the cardiologists have marked out for themselves the domain of diseases of the heart.

