# Erratum

**Published:** 1913-07-05

**Authors:** 


### ERRATUM.

In our last week's issue an accidental omission of a sub-heading took place on p. 383. The words in question should have been inserted at the end of the paragraph on "Delayed Lactose Excretion," and were "Nephritis with Delayed Iodide Excretion." Thus the third paragraph in that column deals with a quite different form of disease from that alluded to in the second paragraph.

